# Leptin and leptin receptor gene polymorphisms and their association with plasma leptin levels and obesity in a multi-ethnic Malaysian suburban population

**DOI:** 10.1186/1880-6805-33-15

**Published:** 2014-06-20

**Authors:** Sook-Ha Fan, Yee-How Say

**Affiliations:** 1 Department of Biomedical Science, Faculty of Science, Universiti Tunku Abdul Rahman (UTAR) Perak Campus, Kampar, Malaysia

**Keywords:** Leptin, Leptin receptor, Single nucleotide polymorphism, Obesity, Malaysia

## Abstract

**Background:**

This study was to investigate the prevalence of single nucleotide polymorphisms (SNPs) in leptin gene *LEP* (A19G and G2548A) and leptin receptor gene *LEPR* (K109R and Q223R) and their association with fasting plasma leptin level (PLL) and obesity in a Malaysian suburban population in Kampar, Perak.

**Methods:**

Convenience sampling was performed with informed consents, and the study sample was drawn from patients who were patrons of the Kampar Health Clinic. A total of 408 subjects (mean age, 52.4 ± 13.7 years; 169 men, 239 women; 190 obese, 218 non-obese; 148 Malays, 177 ethnic Chinese, 83 ethnic Indians) participated. Socio-demographic data and anthropometric measurements were taken, and genotyping was performed using polymerase chain reaction-restriction fragment length polymorphism (PCR-RFLP).

**Results:**

The *LEP* A19G, G2548A and *LEPR* K109R, Q223R variant allele frequencies were 0.74, 0.67 and 0.61, 0.79, respectively. The genotype and allele distributions of these gene variants were significantly different among ethnic groups, but not among body mass index (BMI) classes. Subjects with *LEPR* K109 and Q223 allele had significantly higher systolic blood pressure and adiposity indices after adjustment for ethnicity (higher BMI, total body and subcutaneous fat; lower skeletal muscle percentage). Subjects with *LEPR* 109R allele had lower PLL than their wild-type allele counterparts. The influence of *LEP* A19G and G2548A SNPs on blood pressures, anthropometrics, and PLL was not evident. Interestingly, synergistic effect of the *LEP* and *LEPR* SNPs was observed as subjects homozygous for all four SNPs studied exhibited significantly higher subcutaneous fat and PLL than those with other genotype combinations.

**Conclusions:**

The *LEP* and *LEPR* SNPs in this study may not be an obesity marker among Malaysians in this population, but were associated with ethnicity. Our findings suggest that each of these SNPs contributes to minor but significant variation in obesity-related traits and in combination they display synergistic effects on subcutaneous fat and PLL.

## Background

Obesity is defined as excessive fat accumulation in the body caused by an imbalance of energy between caloric consumption and expenditure [1]. The etiology of obesity is multifactorial as it involves genetic and environmental factors as well as the complex interactions among them [2]. The prevalence of obesity is rising at an alarming rate worldwide to the extent that it is currently considered a pandemic rather than epidemic. The Malaysian Fourth National Health and Morbidity Survey (NHMS IV) conducted in 2011 reported that the prevalence of overweight and obese has increased from 29.1% to 29.4% and 14.0% to 15.1%, respectively [3]. One of the most important routes involved in regulating body weight and energy expenditure is the leptin-melanocortin signaling pathway.

Leptin, an adipocyte-derived hormone, acts as a satiety signal by downregulating orexigenic peptides (neuropeptide Y, agouti-gene related peptide, melanin-concentrating hormone, and orexin) which stimulate appetite and upregulating anorexigenic peptides (alpha-melanocyte stimulating hormone, cocaine and amphetamine-regulated transcripts, and corticotropin-releasing hormone) which inhibit feeding [4]. Leptin exerts its physiological effect by binding to leptin receptor, a single transmembrane protein which belongs to class I cytokine receptor family. Of all the *LEPR* isoforms described so far, the long isoform *LEPRb* is of physiological importance [4]. Upon leptin binding to its receptor, a *LEPRb*/Janus kinase 2 (JAK2) complex is formed, resulting in cross-phosporylation. The tyrosine residue, Tyr1138 on *LEPRb* is important for signal transducer and activator of transcription 3 (STAT3) activation, which activates suppressor of cytokine signaling 3 (SOCS3) expression. This leads to negative inhibition of leptin signaling through Tyr985 and additional sites on JAK2. Mitogen-activated protein kinase (MAPK) and insulin receptor substrate/phosphatidyl-inositol 3’ kinase (PI3K) pathways can be activated following JAK2 phosphorylation [4].

Several polymorphisms of both the genes were studied in different populations for its potential association with obesity and its related complications. Among these variants, the *LEP* A19G and G2548A as well as *LEPR* K109R and Q223R single nucleotide polymorphisms (SNPs) have been studied in detail. These findings were replicated across different populations, revealing contradictory findings. Most literature to date regarding the association between obesity and these polymorphisms are less clear and notably scarce in non-Caucasian populations. The lack of data on this subject emphasizes the need for studies among Malaysians and also across ethnic groups.

In the present study, we determined the prevalence of the *LEP* A19G, *LEP* G2548A, *LEPR* K109R, and *LEPR* Q223R SNPs and its possible association with obesity and its related traits in a Malaysian multi-ethnic suburban population in Kampar, Perak.

## Methods

### Subjects and anthropometric measurements

According to the 2010 population and housing census [5], the population size in Kampar was 88,638 with a ratio of 3:5:1 for the Malays, Chinese, and Indians while the ratio of the subjects in this study was 2:2:1. The higher number of Chinese in the Kampar population census could be due to the inclusion of college/university students (Kolej Tunku Abdul Rahman/Universiti Tunku Abdul Rahman, Perak campus) which are predominantly Chinese. Based on this population size, in order to detect margin of error of 5%, with a confidence level of 95% and a response distribution of at least 50%, a minimum sample size of 383 subjects had to be recruited (Raosoft® Sample size calculator software, Raosoft Inc., US). In this study, the available sample size for statistical analysis was 408 subjects although a total of 481 subjects were recruited in the beginning, owing to blood insufficiency, low concentration of extracted DNA, insufficient remaining DNA sample, missing data, and duplicate cases. The ethnicities of the subjects were self-identified in three given choices: Malay/Peninsular Bumiputera (Orang Asli), Chinese, or Indian. The subjects (169 men, 239 women; 190 obese, 218 non-obese) had a mean age of 52.4 ± 13.7 years and comprised 148 Malays, 177 ethnic Chinese, and 83 ethnic Indians. They were unrelated subjects visiting the Kampar Health Clinic who had fasted overnight prior to the blood collection. Subjects with known medical conditions such as hyperthyroidism, pituitary diseases, chronic liver or renal diseases, acute infection, and hematologic diseases were excluded from the study. Other exclusion criteria were pregnancy, patients undergoing dialysis, body builders or highly trained athletes, people with a fever or swelling, and patients with osteoporosis who have very low bone density.

Anthropometric measurements (systolic and diastolic blood pressures (SBP and DBP, respectively), waist and hip circumference (WC and HC, respectively), weight, height and body compositions such as total body fat (TBF), subcutaneous fat (SF), visceral fat level (VFL), resting metabolism (RM), and skeletal muscle percentage (SM)) were obtained from subjects as previously described [6]. Subjects with a body mass index (BMI) of 27 kg/m^2^ were considered as obese [7].

This study was registered under the National Medical Research Registry (NMRR-09-826-4266) and the protocol was approved by the Medical Research and Ethnics Committee, Ministry of Health Malaysia. All subjects participating in this study signed informed consent forms and all samples were taken in accordance with the Declaration of Helsinki (revised in Seoul, 2008).

### Genotyping

Genomic DNA was extracted from the leucocytes using Wizard® Genomic DNA Purification Kit (Promega Inc., Madison, WI, USA) according to the manufacturer’s protocol. PCR-RFLP was performed according to the conditions adapted from previous studies [8-10]. The products were electrophoresed on agarose gel (2% for the *LEP* variants or on 3% for *LEPR* SNPs). PCR products of three samples with known genotype from each SNP were verified by direct DNA sequencing (First BASE Laboratories Sdn. Bhd., Malaysia).

### Plasma leptin level determination

Plasma leptin level (PLL) was determined using commercially available enzyme-linked immunoabsorbent assay (ELISA) kit (Assaymax Human Leptin ELISA kit, Assaypro, USA) according to the manufacturer’s instructions.

### Statistical analysis

The data were analyzed using Statistical Package for Social Sciences (SPSS) for Windows® Version 17.0 (SPSS Inc, Chicago, IL, USA). Allelic frequencies for each SNP were estimated by gene counting and the distribution of genotypes was tested for Hardy-Weinberg equilibrium using the Chi-square (χ^2^) test. Data for continuous variables were presented as means ± standard deviations (SD) or adjusted means ± standard error of the mean (SEM) and as frequency for categorical variables. The normality of distributions of continuous variables was tested with the Kolmogorov-Smirnov test and variables that were not distributed normally were log-transformed prior to statistical analysis. Genotype and allele frequencies of the SNPs with respect to BMI status, gender, and ethnicity were assessed for association using χ^2^ test while Student’s *t* test or analysis of covariance using general linear model (adjusted for ethnicity) were performed for continuous variables. A *P* value of less than 0.05 was considered to be statistically significant.

Statistical power calculation was performed to ensure the study has adequate sample size to detect the association of genetic variants and obesity and its related traits by using Quanto version 1.2.4 (http://biostats.usc.edu/Quanto.html). The statistical power was set to be 80% (two-sided) at 5% level of significance. Based on the required parameters, the minimum number of subjects needed to achieve 80% power would be 130, 124, 126, and 142 for *LEP* A19G, *LEP* G2548A, *LEPR* K109R, and *LEPR* Q223R, respectively. These sample sizes in the present study were more than adequate when the subjects were analyzed as a whole or separately according to their ethnicity, except for Indians. In this study, there were 148 Malays, 177 Chinese, and 83 Indians. For Indians, their sample size of 83 subjects would only achieve 57% to 63% power for all four SNPs. The overall sample size of 408 subjects in this study would achieve a statistical power of 99%.

## Results and discussion

### Allele frequencies and associations with obesity, ethnicity, and gender

The variant allele frequency (VAF) for *LEP* A19G, G2548A and *LEPR* K109R, Q223R were 0.74, 0.67 and 0.61, 0.79, respectively. The genotype distribution of *LEP* SNPs, but not *LEPR* SNPs, was in Hardy-Weinberg equilibrium. The VAF for all the SNPs except for *LEP* G2548A were higher than that reported by Liew *et al.*[11] in a sample of Malaysian university students, which was conducted as part of a pilot study prior to the present research. The difference in allele frequency could be attributed to the imbalance distribution in gender and ethnicity of the pilot study as majority of the subjects were Chinese women as well as the fewer numbers of obese subjects recruited.

The genotype and allele frequencies of all SNPs were not associated with obesity and gender, but with ethnicity (Table 1). However, the frequency of *LEPR* 109R allele was significantly different among gender. In accordance with most previous studies, this study failed to find an association between the *LEP* A19G polymorphism and obesity as supported by findings in Finnish subjects [8], Italian obese patients [12], Brazilians of European descent [13], Caucasians [14], and an African tribal population group [15]. The *LEP* A19G variant (rs2167270) is a single base transition from A → G at nucleotide position 19 in the 5′ untranslated region (UTR) of exon 1 of the *LEP* gene [16]. As this variant is located within the first untranslated exon of the gene, it is not known how such an alteration might modify protein function [17]. Nevertheless, it has been proposed that this SNP is in disequilibrium with promoter region variation that may have an effect on gene transcription [18]. However, the mechanisms of how the DNA sequence variability in the promoter region influences promoter activity or gene expression are still less clear and merit further investigation.

**Table 1:** Genotype and allele frequencies of LEP and LEPR SNPs according to BMI status, ethnicity, gender

**SNP/Genotype/Allele**	**Obesity status**		**Ethnicity**			**Gender**	
	**Non-obese**	**Obese**	Malay*	**Chinese**	**Indian**	**Male**	**Female**
***LEP *** **A19G**							
AA	12	10	4	11	7	9	13
AG	88	79	52	73	42	63	104
GG	118	101	92	93	34	97	122
*P*	0.968			**0.024**		0.430	
A	112	99	60	95	56	81	130
G	324	281	236	259	110	257	348
*P*	0.906			**0.006**		0.299	
***LEP *** **G2548A**							
GG	28	20	14	17	17	24	24
GA	81	89	59	67	44	62	108
AA	109	81	75	93	22	83	107
*P*	0.140			**0.001**		0.167	
G	137	129	87	101	78	110	156
A	299	251	209	253	88	228	322
*P*	0.443			**< 0.001**		0.978	
***LEPR *** **K109R**							
KK	43	41	27	6	51	28	56
KR	75	76	63	60	28	62	89
RR	100	73	58	111	4	79	94
*P*	0308			**< 0.001**		0.169	
K	161	158	117	72	130	118	201
R	275	222	179	282	36	220	277
*P*	0.174			**< 0.001**		**0.040**	
***LEPR *** **Q223R**							
QQ	14	14	7	4	17	6	22
QR	58	59	49	40	28	50	67
RR	146	117	92	133	38	113	150
*P*	0.524			**< 0.001**		0.084	
Q	86	87	63	48	62	62	111
R	350	293	233	306	104	276	367
*P*	0.269			**< 0.001**		0.093	

All values by Chi-square test, significant at *P* < 0.05 and indicated in bold font.

*LEP,* leptin; *LEPR,* leptin receptor; SNP, single nucleotide polymorphism. ^a^Malay/Peninsular Bumiputra.

According to Li *et al.*[19], this *LEP* G2548A variant was more common in Caucasians than African-Americans within the average-weight groups. The present study found that the variant A allele was higher in Asians, at least among Malays/Peninsular Bumiputras, Chinese, and Indians. Numerous studies have failed to detect an association between this genetic variant and obesity as supported by research among Tunisians [20], Pacific Islanders [21], Spanish [22] and Africans [15]. The *LEP* G2548A SNP (rs7799039) is a G → A transition at nucleotide position -2548 upstream of the ATG start site in the *LEP* gene 5′ promoter region [23]. According to Jiang *et al.*[24], since the *LEP* G2548A polymorphism is not at a conserved region among human, mouse, and rat species, its functional significance is uncertain. On the other hand, this polymorphism is located at the 5′ end of the promoter region of *LEP*[25]. It has been postulated that this remote region might contain inhibitory elements for transcription in adipocytes [26]. Although the *LEP* G2548A polymorphism is close to these elements and putative binding sites, the effect of this SNP on leptin expression remains to be investigated.

The higher VAF for *LEPR* K109R found in this study was confirmed by recent findings of a systematic review and meta-analysis by Bender *et al.*[27]. According to them, there was a strong difference in allelic frequencies between Caucasians and Asians, with Asians showing much higher derived allele frequencies for 109R (76.5% to 84.4% compared to 12.3% to 35.3% in Caucasians). In this study, the genotype and allele distribution of *LEPR* K109R were found to be associated with ethnicity. Similarities were observed in the VAF of this SNP among subjects of the same ethnic backgrounds; specifically ethnic Indians and Chinese in this study with Chinese and Indian nationals. Among ethnic Indians, the VAF was 0.22; in agreement with 0.21 in a study by Murugesan *et al.*[28] which consisted of a local population in Coimbatore, with majority being Tamil Nadu Indians. Indeed, majority of Malaysian Indians in this study comprises of Tamils and Telugus with ancestries tracing back to the Indian subcontinent [29]. On the other hand, the VAF for Chinese was 0.80, in line with 0.81 among Chinese population as reported recently by Lu *et al*. [30]. The subjects in Lu *et al.*’s study were a population of Han Chinese in Shanghai while the Malaysian Chinese subjects in this study comprises mostly of Han Chinese descent. It has been documented that Chinese migrants predominantly originated from Han Chinese from the southern provinces of China, such as Guangdong and Fujian [31]. Therefore, the similar VAF found between both ethnic groups in Malaysia and those in China and India reflect the genetic ancestral origins based on their migration history. With regard to Malays, observation cannot be made as no published data are available for comparison.

The *LEPR* K109R SNP (rs1137100) is an A → G transition in codon 109 (AAG to AGG) at position 326 in exon 4. This causes a conservative change that resulted in a change of amino acid lysine to arginine (Lys/K to Arg/R) [10]. Although this alteration produces amino acid changes and hence may have functional consequences, its change in functionality is not evident. No actual evidence of the possible functional implications of nucleotide alterations in *LEPR* is available and yet it is likely that these variants serve as genetic markers for nearby functional variants which are in linkage disequilibrium with selected markers [32]. Interestingly, as an alternative explanation for variation in allele frequency, Hancock *et al.*[33] reported that associations of some *LEPR* variants (which includes K109R) with climate variables, indicating a role of climate adaptations in the biological mechanisms underlying metabolic phenotypes such as cold adaptation and overweight. The researchers suggest that variants such as *LEPR* K109R are deleterious in hot equatorial climates might be advantageous in colder climates. According to them, *LEPR* K109R is one of the best examples to illustrate the relationship between genetic susceptibility to metabolic disorders and climate adaptations.

The VAF for *LEPR* Q223R reported in the present study supports previous findings of much higher derived allele frequencies for 223R in Asians (80.6% to 95.0% compared to 30.2% to 56.7% in Caucasians) [27]. Indeed, evidence for recent positive natural selection of variants in polymorphic genes including *LEPR* Q223R was described in the major ethnic groups globally, among them Asian populations [34]. It was demonstrated that in many of the putatively selected regions, there is a significant over-representation of genes associated with complex diseases. The present results are in agreement with previous studies which reported non-association with obesity [10,35,36]. However, conflicting results were also reported as this SNP was found to be associated with obesity [13,37,38]. The *LEPR* Q223R SNP (rs1137101) is an A → G transition that causes a non-conservative change which converts a glutamine to an arginine (Gln/Q to Arg/R) in codon 223 (CAG to CGG) at position 668 in exon 6 [10]. *LEPR* Q223R SNP results in changes in charge (neutral to positive) and is located within the leptin-binding region in the extracellular domain of the *LEPR* gene. Thus, changes in amino acid affect all isoforms of the receptor and may be linked to impaired signaling capacity. The fact that *LEPR* Q223R polymorphism in human resembled Q269P mutation in Zucker rat model in terms of proximity and similarity may possibly explain the leptin resistance in human as a result of profound changes in its signaling pathways [37].

Since the distribution of genotypes and alleles of *LEP* and *LEPR* SNPs were significantly different between ethnicities, a separate analysis based on ethnicity was performed. This is to assess its association within each ethnic group as earlier analyses were carried out on a heterogeneous mix of subjects. Within the Malay, Chinese, and Indian ethnic group, there was no significant association between the allele frequencies of all the four SNPs studied and obesity (data not shown). Similarly, the allele frequencies of the SNPs studied were found to be not associated with genders among the Malays and Chinese, respectively. However, among Indians, an association was found between the *LEPR* Q223R allele frequency and genders while no such association was observed for the three other SNPs.

Taken together, these findings demonstrate that it is difficult to explain obesity on the basis of common polymorphisms in the human *LEP* and *LEPR* genes. Possible predisposition to obesity due to different combination of allelic variants from different genes can obscure differences in genotype and allele frequencies between normal and obese subjects, especially when comparison is being done one gene at a time [39]. The absence of association might not necessarily suggest a lack of effect, instead it reflects the complex pathogenesis of obesity which comprises environmental factors in addition to genetic component [40].

### Associations with anthropometric and clinical variables

All the anthropometric and clinical variables assessed were not significantly different among alleles of *LEP* A19G and G2548A (Table 2). This result is in line with a previous study [8] which reported that *LEP* A19G was not associated with any obesity-related variables evaluated, including weight, BMI, fat mass (FM), waist-to-hip ratio (WHR), respiratory quotient, and basal metabolic rate. Likewise, no such association with common obesity-related variables (BMI, FM, WC, WHR, metabolic parameters, and blood pressures) was reported in Romanian [22] or Polish subjects in relation to *LEP* G2548A [41]. This SNP seems to have no effect on the wide range of anthropometric indicators of obesity, at least among the sampled Malaysian population.

**Table 2:** **Means of anthropometric and clinical variables according to *LEP *** **and *LEPR *** **SNPs genotypes and alleles**

**Anthropometric and clinical variables**	**Mean ± SEM**											
	***LEP *** **A19G**			***LEP *** **G2548A**			***LEPR *** **K109R**			***LEPR *** **Q223R**		
	**Alleles**			**Alleles**			**Alleles**			**Alleles**		
	**A**	**G**	***P***	**G**	**A**	***P***	**K**	**R**	***P***	**Q**	**R**	***P***
SBP (mmHg)	137.56 ± 1.47	140.05 ± 0.87	0.146	139.75 ± 1.32	139.24 ± 0.91	0.751	141.85 ± 1.21	137.84 ± 0.96	**0.010**	144.00 ± 1.62	138.17 ± 0.84	**0.001**
DBP (mmHg)	80.53 ± 0.74	81.27 ± 0.43	0.387	81.79 ± 0.66	80.74 ± 0.45	0.187	82.06 ± 0.60	80.45 ± 0.48	**0.040**	82.08 ± 0.81	80.81 ± 0.42	0.166
Pulse rate (bpm)	73.32 ± 0.90	74.32 ± 0.53	0.335	73.74 ± 0.80	74.22 ± 0.56	0.621	75.14 ± 0.74	73.88 ± 0.59	0.065	74.98 ± 0.99	73.82 ± 0.51	0.298
WC (cm)	90.52 ± 0.80	91.16 ± 0.47	0.488	91.10 ± 0.71	90.95 ± 0.50	0.862	92.95 ± 0.65	89.74 ± 0.52	**< 0.001**	92.43 ± 0.88	90.61 ± 0.46	0.068
WHR	0.89 ± 0.01	0.90 ± 0.003	0.231	0.90 ± 0.01	0.89 ± 0.003	0.341	0.90 ± 0.004	0.89 ± 0.004	**0.039**	0.89 ± 0.01	0.89 ± 0.003	0.973
Weight (kg)	69.29 ± 1.02	69.87 ± 0.60	0.625	70.11 ± 0.91	69.54 ± 0.63	0.611	71.42 ± 0.84	68.63 ± 0.67	**0.010**	70.95 ± 1.13	69.39 ± 0.58	0.223
BMI (kg/m^2^)	27.01 ± 0.35	27.22 ± 0.20	0.606	27.15 ± 0.31	27.18 ± 0.21	0.940	28.05 ± 0.28	26.60 ± 0.23	**< 0.001**	27.85 ± 0.38	26.99 ± 0.20	**0.047**
TBF^a^ (%)	33.27 ± 0.48	33.31 ± 0.28	0.740	33.29 ± 0.43	33.31 ± 0.30	0.808	34.46 ± 0.39	32.56 ± 0.31	**0.001**	34.70 ± 0.53	32.93 ± 0.27	**0.007**
SF^a^ (%)	27.36 ± 0.57	27.14 ± 0.33	0.904	27.14 ± 0.50	27.22 ± 0.35	0.913	28.72 ± 0.46	26.22 ± 0.37	**< 0.001**	28.87 ± 0.62	26.75 ± 0.32	**0.005**
VFL^a^ (%)	11.79 ± 0.43	12.44 ± 0.25	0.188	12.16 ± 0.38	12.32 ± 0.27	0.636	13.06 ± 0.35	11.76 ± 0.28	**0.006**	12.92 ± 0.47	12.09 ± 0.25	0.076
RM (kcal)	1448.53 ± 18.06	1459.04 ± 10.63	0.617	1462.09 ± 16.09	1453.54 ± 11.16	0.663	1468.24 ± 14.83	1448.68 ± 11.82	0.308	1457.77 ± 19.95	1455.94 ± 10.31	0.935
SM^a^ (%)	25.09 ± 0.28	25.05 ± 0.17	0.919	25.19 ± 0.25	24.99 ± 0.17	0.536	24.58 ± 0.23	25.36 ± 0.18	**0.005**	24.39 ± 0.31	25.24 ± 0.16	**0.010**
Leptin^a^ (ng/mL)^b^	33.48 ± 39.10	27.31 ± 35.29	0.325	32.40 ± 37.75	27.22 ± 35.63	0.053	32.34 ± 42.21	26.70 ± 31.96	**0.010**	30.06 ± 36.68	28.60 ± 36.34	0.058

All values by univariate analysis of variance (General Linear Model) and adjusted for covariate ethnicity, significant at *P* < 0.05 and indicated in bold font. Values presented as adjusted mean ± SEM (estimated marginal means ± standard error of the mean).

^a^Values were log transformed before analysis.

^b^Leptin values were analyzed by Student’s *t*-test and presented as mean ± SD (standard deviation).

BMI, body mass index; DBP, diastolic blood pressure; RM, resting metabolism; SBP, systolic blood pressure; TBF, total body fat; SF, subcutaneous fat; SM, skeletal muscle; VFL, visceral fat level; WC, waist circumference; WHR, Waist-to-hip ratio.

For *LEPR* K109R, subjects carrying the wild-type K allele showed a significant trend of higher means for blood pressures and all anthropometric measurements, except for pulse rate and RM but lower SM. These results are in accordance with a number of studies but in contrast to many others. Rosmond *et al.*[42] found that variant R homozygotes had lower SBP (10.0 mmHg) and DBP (7.8 mmHg), BMI, and abdominal sagittal diameter. This observation is in agreement with the present study where carriers of R allele also showed lower blood pressures (SBP and DBP) and BMI. However, the small differences detected for SBP (4.01 mmHg) and DBP (1.61 mmHg) may not be of clinical significance. Lu *et al.*[30] also reported that KK homozygotes had higher SBP in men but this variant was not associated with obesity-related traits (weight, BMI, WC, WHR, insulin, lipids, uric acid, and DBP).

The anthropometric measurements evaluated in this study were not significantly different between the alleles of *LEPR* Q223R with the exception of SBP, BMI, TBF, SF, and SM. Carriers of the variant R allele had significantly lower mean SBP of 5.83 mmHg and mean BMI of 0.86 units compared to those with wild-type Q allele. With respect to blood pressures, this is in agreement with findings by Rosmond *et al.*[42], which found that RR homozygotes also exhibited lower SBP and DBP (7.6 and 5.7 mmHg, respectively) compared to QQ homozygotes, while no association was found for BMI, WHR, and abdominal sagittal diameter. According to the researchers, this result suggests that leptin induces hypertension through the leptin receptor by activation of central sympathetic nervous system and therefore it is postulated that hypertension is absent with a malfunction central leptin receptor. In this study, it was found that carriers of Q allele had higher adiposity indices (BMI, TBF, and SF). Similarly, QQ homozygotes had on average 64 cm^2^ more total abdominal fat than those carrying R allele [43].

Nevertheless, present results of wild-type *LEPR* K109 allele carriers with higher adiposity indices (WC, WHR, weight, BMI, TBF, SF, and VFL) but lower SM than carriers of variant R allele indicates predisposition to central obesity with higher fat levels and lower muscle mass in those carrying the wild-type allele rather than variant allele. The same observation was seen with the wild-type *LEPR* Q223 allele (higher adiposity indices of BMI, TBF, and SF). This would explain even though these variants (R allele in both SNPs) are prevalent in the sampled population (VAF of 0.61 for *LEPR* K109R and 0.79 for *LEPR* Q223R), no significant association with obesity was found. Since these variants are known to be in higher frequency in Asians compared to other populations, it appears that the sequence variation were in favor (or detrimental) to them in this context some 6,000 to 8,000 years ago [27,44]. As this period coincides with the introduction of agriculture in Asia, it is hypothesized that the *LEPR* gene could be considered a ‘thrifty’ gene, which promotes better fat storage during food abundance and ensuring survival during periods of famine [45].

With respect to PLL, the influence of *LEP* A19G and G2548A and *LEPR* Q223R SNPs, in the present study was not evident; consistent with studies that found no association in *LEP* A19G [12,14], *LEP* G2548A [14,15], and *LEPR* Q223R [41,42]. For *LEPR* K109R SNP, carriers of K allele had higher PLL of 5.64 ng/mL, in agreement with a study by Wauters *et al.*[43], where the KK homozygotes exhibited higher PLL than R carriers in a small subgroup of post-menopausal women. However, the recessive effect of the R allele was also observed, where carriers of the R allele of *LEPR* K109R gene showed increased PLL compared to non-carriers in weight gainers among young Dutch adults [46]. Despite these discrepancies, this implies the existence of genotype-specific effects in the coordination of circulating leptin with regard to active fat deposition [47]. In support of this, a functional study described that this common polymorphism of *LEP* G2548A influenced leptin expression and consequently alter plasma leptin levels, possibly at the transcriptional level [48].

### Relationships between genotype combinations with anthropometric and clinical variables

Mutations in the mouse *LEP* gene homozygous for the *ob/ob* phenotype resulted in the absence of leptin production. However, the homologous mutation in the *LEP* gene in humans has not been reported [49]. The *LEP* gene sequence is highly conserved, being 84% identical with the mouse protein [50]. The first case of human mutation in the leptin gene was reported in 1997 for homozygous frameshift mutation in two children with the same consanguineous pedigree [51]. Severe obesity due to single mutation in the *LEP* gene is so rare that only 12 human cases were reported to date [52]. On the other hand, the *db/db* and Zucker *fa/fa* mutations in chromosome 4 of the mouse cause severe obesity which is irreversible by leptin treatment. The human *LEPR* gene is 78% homologous to the mouse gene [53]. Hence, the severity of phenotype caused by *LEPR* mutations in human would be similar to those observed in mouse models. However, similar to leptin, such mutations are very infrequent in humans. Instead, *LEPR* variants which do not cause such extreme loss of function are more commonly reported [54]. When analyzed individually, only certain SNPs were associated with some of the obesity-related traits such as blood pressures, anthropometric variables, and PLL.

However when analyzed together, it is interesting to note that subjects homozygous for all four SNPs studied exhibited significantly higher SF and PLL than those with other genotype combinations (Table 3). To the best of our knowledge, the synergistic effect of these *LEP* and *LEPR* genotype combinations on SF and PLL has not been reported elsewhere previously. In a Chinese population, Lu *et al.*[30] found that a combination of SNPs in the *LEP* gene 3′ flanking region, *LEPR* K109R and *LEPR* K656N is associated with significantly higher BMI. According to Bouchard *et al.*[55], the contribution of a single gene may not necessarily cause a significant phenotypic variance. However, in combination, they may account for a significant part of the variation, leading to predisposition to obesity. This is particularly true for a polygenic disease such as obesity, where a single gene polymorphism is likely to exert a minor influence on the observed phenotype.

**Table 3:** The relationship between genotype combinations with blood pressures, anthropometric variables, and plasma leptin levels

**Variable**	**Mean ± SD**		
	**Subjects homozygous for all four SNPs**^**a**^	**Subjects not homozygous for all four SNPs**	***P***
	**(n = 70)**	**(n = 338)**	
SBP (mmHg)	137.40 ± 19.83	139.83 ± 22.23	0.398
DBP (mmHg)	81.55 ± 10.51	80.98 ± 10.78	0.688
Pulse rate (bpm)	74.48 ± 12.60	73.98 ± 13.10	0.768
WC (cm)	90.94 ± 12.50	91.01 ± 11.51	0.966
HC (cm)	101.20 ± 11.09	102.13 ± 9.91	0.484
WHR	0.90 ± 0.08	0.89 ± 0.08	0.457
Height (cm)	161.11 ± 8.54	159.81 ± 9.39	0.283
Weight (kg)	70.38 ± 16.34	69.59 ± 14.63	0.686
BMI (kg/m^2^)	27.04 ± 5.57	27.20 ± 5.00	0.813
TBF^b^ (%)	31.92 ± 6.53	33.59 ± 6.98	0.131
SF^b^ (%)	25.25 ± 7.84	27.60 ± 8.20	**0.045**
VFL^b^ (%)	12.64 ± 6.87	12.19 ± 6.23	0.672
RM (kcal)	1,491.96 ± 282.82	1,448.95 ± 258.76	0.214
SM^b^ (%)	25.78 ± 3.82	24.91 ± 4.08	0.084
Leptin^b^ (ng/mL)	21.22 ± 27.96	30.50 ± 37.77	**0.015**

All values by Student’s *t*-test, significant at *P* < 0.05 and indicated in bold font.

^a^Subjects with homozygous mutated genotypes for all four SNPs: GG for *LEP* A19G, AA for *LEP* G2548A, RR for both *LEPR* K109R and *LEPR* Q223R.

^b^Values were log transformed before analysis.

### Study limitations

Inconsistency of the results from this study may be due to sample size, sampling method, and restriction to a certain area which could have a risk of sampling bias. With regard to the moderate size of the screened population, future studies should involve larger sample size of Malaysians from all the states with a more balanced age groups, ethnicities, and genders to better reflect the Malaysian population. Another limitation is this study did not take into account certain variables found by previous studies to be significantly associated with *LEP* and *LEPR* SNPs such as biochemical parameters like serum lipids, glucose, and insulin. Hence, the impacts of these parameters should be considered in future studies.

## Conclusions

The present results show that *LEP* A19G, *LEP* G2548A; *LEPR* K109R and *LEPR* Q223R SNPs are unlikely to be a relevant obesity marker among Malaysians, at least among the Kampar suburban population, but were associated with ethnicity. Findings from this study suggest that each of these SNPs contributes to minor but significant variation in obesity-related phenotypes. The *LEPR* K109R gene variants also influence PLL in Malaysian subjects. Synergistic effect of *LEP* and *LEPR* genotype combinations on SF and PLL was observed.

## Abbreviations

BMI: Body mass index; DBP: Diastolic blood pressure; FM: Fat mass; HC: Hip circumference; JAK2: Janus kinase 2; *LEP*: Leptin gene; *LEPR*: Leptin receptor gene; PLL: Plasma leptin level; PCR-RFLP: Polymerase chain reaction-restriction fragment length polymorphism; RM: Resting metabolism; SBP: Systolic blood pressure; SD: Standard deviation; SEM: Standard error of the mean; SF: Subcutaneous fat; SM: Skeletal muscle; SNP: Single nucleotide polymorphism; TBF: Total body fat; VAF: Variant allele frequency; VFL: Visceral body level; WC: Waist circumference; WHR: Waist-to-hip ratio.

## Competing interests

The authors declare that they have no competing interests.

## Authors’ contributions

SHF carried out the studies, analyzed data, and drafted the paper. YHS participated in the study design. Both authors read and approved the final manuscript.
